# The HOPE Project (Helping Older People Engage): Relevance to Upstream Suicide Prevention

**DOI:** 10.1093/geroni/igaa057.2118

**Published:** 2020-12-16

**Authors:** Kim Van Orden, Yeates Conwell, Ben Chapman, Geoff Williams, Siilvia Sörensen, Jody Rowe

**Affiliations:** 1 University of Rochester School of Medicine and Dentistry, Rochester, New York, United States; 2 University of Rochester, Rochester, New York, United States; 3 URMC, Rochester, New York, United States; 4 Lifespan of the Greater Rochester Area, Rochester, New York, United States

## Abstract

The HOPE Project is an ongoing RCT testing whether Senior Corps volunteering for lonely older adults (age 60+) leads to reduced loneliness and improved quality of life—outcomes associated with suicide in later life. We have randomly assigned 130 participants to 12-months of volunteering or active control. We will describe the trial as well as baseline characteristics of participants that may predict non-compliance with volunteering/control. We found no difference between conditions nor demographic characteristics (age, gender) on non-compliance. Participants demonstrated wide variability in depression at baseline (PROMIS t-score range 38.9 to 71.4) and 18% reported suicide ideation; neither were associated with compliance (p>.20). These preliminary findings indicate that those with more severe mental health symptoms were equally willing/able to engage in volunteering as those without depression and suicide ideation. Volunteering is a highly scalable intervention (given nationwide Senior Corps infrastructure) that may function as upstream suicide prevention.

